# Lack of Association of Childhood Partial Epilepsy with Brain Derived Neurotrophic Factor Gene

**DOI:** 10.1100/2012/414797

**Published:** 2012-05-01

**Authors:** Aycan Unalp, Elcin Bora, Tufan Cankaya, Ozlem Giray Bozkaya, Derya Ercal, Aysel Ozturk, Ayfer Ulgenalp

**Affiliations:** ^1^Dr. Behcet Uz Child Disease and Pediatric Surgery Training and Research Hospital, Montro, Izmir, Turkey; ^2^Department of Medical Genetics, Faculty of Medicine, Dokuz Eylul University, Balcova, 35340 Izmir, Turkey; ^3^Department of Pediatrics, Division of Genetics, Dokuz Eylul University, Balcova, 35340 Izmir, Turkey

## Abstract

Brain-derived factor (BDNF) is a member of neurotrophin family and is localized and upregulated in areas implicated in epileptogenesis. Several lines of evidence make the BDNF gene a plausible candidate gene for predisposition to epilepsy. In this study, we tested that BDNF might be involved in the etiology of childhood PE. To assess whether BDNF gene C270T polimorphism could be implicated in vulnerability to PE, we conducted a case-control association analysis (112 partial epileptic and 100 controls) in Turkish children. Epileptic children were divided into two groups: 1—idiopathic (*n* = 85) and 2—symptomathic epilepsy (*n* = 27). There was no significant difference in genotypic distribution and allelic frequencies of the BDNF gene C270T polimorphism between the PE and control groups. However, the BDNF gene TT genotype was more frequently seen in the epileptic children (15 versus 11 patients, resp.). Interestingly, in the epilepsy group, both two children with TT genotype have posttraumatic epilepsy. The data indicate a possible association with the 270T genotype of the BDNF gene with a posttraumatic epilepsy. To draw any conclusion, further studies using larger sample sizes should be carried out in various ethnic populations in childhood epilepsies.

## 1. Introduction

Epilepsy is a condition or a group of conditions involving transient disturbances in cerebral function caused by abnormal neuronal discharges and characterized by recurrent seizures. Epilepsy is divided into idiopathic, cryptogenic, and symptomatic forms. Among them, idiopathic epilepsy means that the primary etiology of epilepsy is believed to be genetic, and no any underlying pathological disturbance exists [1]. Recent molecular works have suggested that both generalized and focal idiopathic epilepsy are caused by a genetic defect [2]. Epilepsy-associated genes largely involve ion channels, containing voltage- or ligand-gated channels. In addition, nonion channel genes are also identified in response to some epilepsies. Partial epilepsy (PE) has been largely considered as an environmental-dependent disease in the past; however, a set of works suggest that PEs also show a genetic factor in underlying pathogenesis [2].

Brain-derived neurotrophic factor (BDNF), a small dimeric protein and a member of the neurotrophic factor, is expressed widely throughout in the developing brain. Various animal experiments as well as clinical studies have shown that BDNF regulates neuronal morphology, synaptogenesis, and neuroprotective effects in diverse areas of the CNS during development [3]. Several lines of evidence make the BDNF gene a plausible candidate gene for predisposition to epilepsy. BDNF regulates neuronal survival, growth, and connectivity during development and participates in plasticity and maintenance of neurons throughout adulthood [4]. Increased BDNF levels lead to hyperexcitability both *in vivo* and *in vitro* and exogenous BDNF induces spontaneous seizures in rats [5, 6]. Furthermore, BDNF is upregulated by limbic seizures in animal models [7, 8] and in patients with epilepsy [9, 10]. Variations in the BDNF gene might alter function and neurotrophic effects of the BDNF protein, thus ultimately predisposing individuals to the development of epilepsy. In search of a specific genetic factor that may enhance or inhibit posttraumatic development of epileptogenesis, Peltola et al. [11] recently suggested a cytokine gene polymorphism as a possible candidate. Furthermore, Kanemoto et al. [12] reported significant correlation between the C240T polymorphism in the BDNF gene and PE in the Japanese population (*P* = 0.003). As indicated by Lohoff et al. [13], the C/T polymorphism at position 240 in the noncoding region of the BDNF gene (GenBank accession number NM001709) corresponds to the 270C/T polymorphism described by Kunugi et al. [14]. Because the BDNF C270T nomenclature is much more widely used [15–20] than C240T, we refer to this polymorphism as C270T.

In our previous study [21], we investigated BDNF serum concentration in a group of epileptic children and healthy controls, and we did not find a significant difference between the two groups. On the basis of this experience, we further tried to evaluate whether BDNF C270T polymorphism is a useful marker for predicting susceptibility to PEs in children.

## 2. Patients and Methods

This study included all outpatients of the Dr. Behçet Uz Child Disease and Pediatric Surgery Training and Research Hospital-Pediatric Neurology Polyclinic from 2008 to December 2010 who gave their informed consent. The patient group consisted of 112 children, 62.5% males. The control group (*n* = 100) comprised unrelated neurologically normal individuals. Diagnoses were carried out from seizure symptomatology (Commission on Classification and Terminology of the International League Against Epilepsy, 1989), and those who showed focal EEG abnormalities or focal lesions on imaging studies but did not have corresponding seizure symptoms suggestive of focal origin were excluded. Study group was divided into two categories: those with PE and MRI evidence (symptomatic epilepsy) (*n* = 27), those with PE essentially normal MRI findings (idiopathic epilepsy) (*n* = 85).

Genomic DNA was extracted from peripheral whole blood using NucleoSpin Blood kit according to the manufacturer's instructions (Macherey-Nagel, Germany). The C270T polymorphism of intron 1 of the 5_-UTR sequence was first reported by Kunugi et al. (GenBank accession no. X60202) [14]. The genotyping procedure consisted of polymerase chain reaction (PCR) amplification and SNP detection of the C270T variant using the following pair of primers: BDNF-F: 5′-CAG AGG AGC CAG CCC GCT GCG-3′ and BDNF-R: 5′-CTC CTG CAC CAA GCC CCA TTC-3′ more than direct sequencing. PCR amplification was carried out in a volume of 25 *μ*L containing 100 ng DNA; 100 *μ*M of each dATP, dCTP, dGTP, and dTTP; 1x buffer (50 mM NaCl, 10 mM Tris-HCl, 10 mM MgCl_2_, 1 mM DTT, pH_7.5 at 25°C); 5x of Tuneup solution; 25 nM of each primer; 1 U of Taq polymerase (Nanohelix, South Korea). The amplification protocol conditions selected were as follows: initial denaturation at 95°C for 3 min, followed by 35 cycles of denaturation at 94°C for 30 s, annealing at 60°C for 45 s, extension at 72°C for 45 s, and a final extension at 72°C for 7 min. PCR products were purified using PureHelix PCR Purification Kit according to the manufacturer's instructions (Nanohelix, South Korea) and subjected to automatic sequence analysis (automated sequencer ABI 3130; Applied Biosystems, CA 94404, USA) by BigDye terminator reaction according to the supplier's instructions (ABI Prism BigDye Terminator Cycle Sequencing Ready Reaction Kits Version 3.1; Applied Biosystems, CA 94404, USA) using BDNF-F: 5′-CAG AGG AGC CAG CCC GCT GCG-3′ primer. The obtained sequences were analyzed using BioEdit software, version 7.0.5.3 (http://www.mbio.ncsu.edu/bioedit/bioedit.html).

Statistical analysis was performed by using *χ* ^2^ test. A value of *P* < 0.05 was considered statistically significant.

## 3. Results

PE group consists of 112 children with ages ranging from 2 to 17 years and an average age of 10.4 (SD: 3.18) years, those with 85 (75.9%) children in the idiopathic epilepsy group and 27 (24.1%) children in the symptomatic group. One hundred control children (51 females, 49 males) with mean age 10.8 (SD: 3.15) were enrolled in the study.

BDNF 270T allele was found to be in 15 (13%) epileptic and 11 (11%) control children. The BDNF 270T allele variation was not significantly different between the two groups (*P* = 0.748). Comparison of each group by sex and BDNF 270T allele frequencies is presented in Table 1.

Table 3 shows genotype distribution for the C270T polymorphism of the BDNF gene among patients with epilepsy and control group. However, the frequency of genotype was not significantly different from that in epilepsy or that in the controls (*P* = 0.379). Interestingly, in the epilepsy group, both two children with TT genotype have posttraumatic epilepsy. Further, for the genotype frequencies of BDNF gene polymorphism, all two patient subgroups (idiopathic/symptomatic epilepsy), epileptic children with mental retardation and cerebral palsy showed similar distribution of the 270C/T allele (Table 2).

The determination of allelic frequencies of the BDNF gene for idiopathic and symptomatic epilepsy patients is presented in Table 3. The most frequent variant is C allele, in both groups. T allele frequency is higher in the idiopathic group (11 and 4 children, resp.; *P* = 0.792). There was no statisticaly significant difference between the epilepsy and control groups for allele frequency of BDNF gene polymorphism (*P* = 0.738).

## 4. Discussion

Various mechanisms and associated genes have been shown to role in the epileptogenesis, and currently there are many problems in the treatment of epilepsy a need to new solutions. A genetic contribution to the development of PE as a sequel of acquired focal brain disorder has been suggested for a long time. Rimoin and Metrakos [22] concluded that genetic factors may regulate susceptibility to seizures, not only by enhancing the vulnerability to epileptiform activity in some individuals, but also by a protective effect in others. This genetic predilection, which has been suggested but remained unspecified for more than four decades, is now being rapidly elucidated with the recent advances in gene analysis [23]. With a lifetime incidence of up to 3%, epilepsy is one of the most common neurological disorders. Idiopathic epilepsies are assumed to be mainly genetic in origin, present with normal brain imaging, and are estimated to represent up to 47% of all epilepsies [24]. Genetic alterations can also cause symptomatic epilepsies, as in cortical malformations.

In our study, we did not find any significant difference between the PE and control groups for the BDNF C270T variation. To our knowledge, this was the first study which investigates this polymorphism in the childhood epilepsy. Kanemoto et al. [12] detected an association between the 270T allele polymorphism and partial epilepsy, in a group of Japanese adult patients. In contrast, Lohoff et al.'s study [13] failed to document an association between C270T polimorphisim and temporal lobe epilepsy. It was thought that this discrepancy for the results has been based on the ethnic differences.

In search of a polymorphism in the BDNF gene an associated study by Kunugi et al. [14] found that the frequency of the mutated type (T270) was significantly more common in patients with Alzheimer's disease than in controls, suggesting a possible association of the T allele with a low production of BDNF. Nanko et al. [25] detected significant association between the T allele and schizophrenia in Japanese subjects. Watanabe et al. [26] observed similar tendency for the T allele frequency although the difference was not statistically significant. This diverseness may be due to the different ethnicity of the participants or the small sample sizes.

Various studies have shown that BDNF increased neuronal excitability and is the ability to potentiate glutamatergic synaptic transmission. BDNF is highly localized and upregulated in areas implicated in epileptogenesis. Indeed, it is perhaps no coincidence that BDNF-immunoreactive fibers innervate the regions most vulnerable in temporal lobe epilepsy [27]. BDNF mRNA expression within the central nervous system has been known to be tightly regulated by neuronal activity. Seizure activity increases the expression of BDNF mRNA and protein, and recent studies have shown that interfering with BDNF signal transduction inhibits the development of the epileptic state *in vivo*. Thus, transgenic mice with decreased BDNF have a higher seizure threshold, and overexpression of BDNF facilitates seizures [28]. Particularly interesting is the evidence that BDNF expression increases after seizures and after other insults as well. These results contribute to epileptogenesis. This raises the possibility of designing therapies for this disorder that may be both anticonvulsant and antiepileptogenic. New evidence from human tissue resected from intractable epileptics shows that BDNF expression and action in human tissue are consistent with those in the rat, supporting the hypothesis that BDNF may play a role in human temporal lobe epilepsy. Besides, the localization studies have shown in hippocampal slices from TLE patients that BDNF exposure potentiates granule cell excitation and impairs granule cell inhibition [29]. Both the amplitude and frequency of excitatory postsynaptic currents were increased, and there was a decrease in amplitude of evoked inhibitory postsynaptic currents. Subsequent studies demonstrated additional effects of BDNF in hippocampus, such as decreased *γ*-aminobutyric acid (GABA)ergic transmission, phosphorilation of N-methyl-d-aspartate (NMDA) receptors, and actions at sodium channels [30–32]. These works strongly support a role of BDNF in human epilepsy and raise the possibility that interference with BDNF expression or BDNF action may be a therapeutic strategy for treating epilepsy.

Most of the epilepsy-associated genes identified so far encode ion channels. Interestingly, those proteins encoded by the nonion channel genes have been suggested to interact with ion channels. This is conceivable from a pathophysiological point of view, as ion channels provide the basis for both the electrophysiological excitability of neuronal cell membranes and the communication between neurons. Also, most anticonvulsant drugs in clinical use today modulate different types of ion channels [33]. Genetic techniques have been developing rapidly. Whole genome approaches now allow the detection of common genetic variants in complex genetic syndromes. Chip technology also makes this possible. The next generation of sequencing techniques is also developing. This will enable more rapid, more effective, and cheaper genotyping, so that we expect to figure out many more genetic defects in epilepsies and other diseases to be unravelled in the near future. To translate these findings and use them for daily clinical practice is another issue. However, knowledge of the genetic defects can be relevant for diagnosis and prognosis and may even have an impact on therapy, for example, in early use of stiripentol in severe myoclonic epilepsy of infancy [34]. The knowledge of genetic defects and their underlying mechanisms can give rise to new therapeutic strategies in epilepsies in general.

The major hypotheses for the functional effects of insult-induced neurotrophin changes are protection against neuronal damage and stimulation of sprouting and synaptic reorganization; therefore, a BDNF gene polymorphism is an ideal candidate for a genetic predisposition that regulates development of epileptogenesis following insult to the brain [35]. The BDNF polymorphism at position 270 lies within the proBDNF sequence. It may affect the production of mature and biologically active BDNF by altering proBDNF processing [36]. Lower levels of mature BDNF may reduce the amount of protection of neurons from toxic environmental stimuli such as head trauma. Thus, the BDNF polymorphism at position 270 has the potential to affect the activity of extracellular proBDNF activity as well, which may also contribute to development of chronic PE. In our study, although not statistically significant, two patients with PE have TT allele, and both of them have posttraumatic epilepsy.

There was an idiopathic PE dominance, in our patient population. Some PEs such as Rolandic epilepsy have long been known to have a strong genetic contribution; however, the strictly age-dependent nature as well as family history associated with that condition presents a unique clinical picture suggestive of a close kinship with idiopathic generalized epilepsies. Also, many sporadic PE cases cannot be explained as a single-gene disease, because they seem as a polygenic or multifactorial. Nevertheless, genetic and acquired risk factors are frequent in PE patients [12]. In summary, although we cannot find a signicant difference, we thought that BDNF gene T allele frequency may enhance the susceptibility of posttraumatic epilepsy and cause chronic epilepsy in genetically predisposed patients, but further studies are warranted with a larger sample size.

## Figures and Tables

**Table 1:** Comparison of sex and frequency of BDNF 270T allele in study groups.

	Epilepsy group (*n* = 112)	Control group (*n* = 100)
Sex		
Female	42	51
Male	70	49
BDNF-T(+)	15*	11
BDNF-T(−)	97	89

*Two of these patients carry 2 BDNF-T alleles (BDNF T/T) (*P* = 0.748, *χ* ^2^ = 0.10).

**Table 2:** Genotype frequencies of BDNF gene polymorphism in the epilepsy, control, mental retardation, and cerebral palsy subjects.

	CC	CT	TT	Total
Control group	89 (89%)	11 (11%)	0	100
Epilepsy group	97 (86.6%)	13 (11.6%)	2 (1.8%)*	112
Idiopathic	74 (87%)	10 (11.8%)	1 (1.2%)	85
Symptomatic	23 (85.2%)	3 (11.1%)	1 (3.7%)**	27
Mental retardation	18 (90)	2 (10%)	0***	20
Cerebral palsy	7 (87.5%)	1 (12.5%)	0****	8

*χ ^2^ = 1.94, *P* = 0.379, **χ ^2^ = 0.749, *P* = 0.688, ***χ ^2^ = 1.107, *P* = 0.575, ****χ ^2^ = 0.325, *P* = 0.85.

**Table 3:** Allele frequencies of BDNF gene polymorphism.

C240T	C allele	T allele	*P*
Control	189 (94.5%)	11 (5.5%)	*P* = 0.738, *χ* ^2^ = 0.11
Epilepsy	207 (93.2%)	15 (6.8%)	
Idiopathic	158	11	*P* = 0.792, *χ* ^2^ = 0.07
Symptomatic	49	4	
